# Structural connectivity networks in Alzheimer’s disease and Lewy body disease

**DOI:** 10.1002/brb3.2112

**Published:** 2021-04-01

**Authors:** Kyoungwon Baik, Jin‐Ju Yang, Jin Ho Jung, Yang Hyun Lee, Seok Jong Chung, Han Soo Yoo, Young H. Sohn, Phil Hyu Lee, Jong‐Min Lee, Byoung Seok Ye

**Affiliations:** ^1^ Department of Neurology Yonsei University College of Medicine Seoul Korea; ^2^ Department of Biomedical Engineering Hanyang University Seoul Korea

**Keywords:** alzheimer's disease, graph analysis, lewy body disease, structural connectivity

## Abstract

**Objective:**

We evaluated disruption of the white matter (WM) network related with Alzheimer's disease (AD) and Lewy body disease (LBD), which includes Parkinson's disease and dementia with Lewy bodies.

**Methods:**

We consecutively recruited 37 controls and 77 patients with AD‐related cognitive impairment (ADCI) and/or LBD‐related cognitive impairment (LBCI). Diagnoses of ADCI and LBCI were supported by amyloid PET and dopamine transporter PET, respectively. There were 22 patients with ADCI, 19 patients with LBCI, and 36 patients with mixed ADCI/LBCI. We investigated the relationship between ADCI, LBCI, graph theory‐based network measures on diffusion tensor images, and cognitive dysfunction using general linear models after controlling for age, sex, education, deep WM hyperintensities (WMH), periventricular WMH, and intracranial volume.

**Results:**

LBCI, especially mixed with ADCI, was associated with increased normalized path length and decreased normalized global efficiency. LBCI was related to the decreased nodal degree of left caudate, which was further associated with broad cognitive dysfunction. Decreased left caudate nodal degree was associated with decreased fractional anisotropy (FA) in the brain regions vulnerable to LBD. Compared with the control group, the LBCI group had an increased betweenness centrality in the occipital nodes, which was associated with decreased FA in the WM adjacent to the striatum and visuospatial dysfunction.

**Conclusion:**

Concomitant ADCI and LBCI are associated with the accentuation of LBCI‐related WM network disruption centered in the left caudate nucleus. The increase of occipital betweenness centrality could be a characteristic biologic change associated with visuospatial dysfunction in LBCI.

## Graphical abstract

We investigated structural connectivity in Alzheimer's disease (ADCI), Lewy bodies disease (LBCI), and mixed disease. LBCI, especially with ADCI, was associated with disintegration of white matter network.

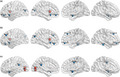

## 1 HIGHLIGHTS

LBCI, especially with ADCI, was associated with disintegration of WM network.

LBCI was associated with decreased nodal degree in the left caudate.

Pure LBCI group had an increased betweenness centrality in the left occipital lobe.

## 2 INTRODUCTION

Alzheimer's disease (AD) and dementia with Lewy bodies (DLB) are the first and second most common causes of degenerative dementia, respectively. Cognitive impairment is also common in patients with Parkinson's disease (PD), which comprises Lewy body (LB) disease (LBD) together with DLB. Previous autopsy studies have shown that LBD and AD pathologies frequently co‐occur (Hamilton, 2000; Irwin et al., 2017). In vivo amyloid imaging and dopamine transporter imaging, which have contributed to more accurate antemortem diagnosis of AD and LBD, also revealed frequent co‐occurrence of the two diseases in cognitively impaired patients (P. Donaghy et al., 2015; Gomperts et al., 2008; S. W. Kang et al., 2019).

White matter (WM) disruption has been observed in AD and LBD, widespread regions involving the medial temporal regions (Firbank et al., 2016; Kantarci et al., 2010; Nedelska et al., 2015) and parieto‐occipital regions (Kantarci et al., 2010; Nedelska et al., 2015), respectively. Although previous studies compared WM connectivity in AD and LBD, only very few studies have considered mixed disease of AD and LBD (P. C. Donaghy et al., 2020; Nedelska et al., 2015). To the best of our knowledge, it remains unknown how AD and LBD are related to WM network disruption.

Graph theory is a mathematical method to analyze complex networks with a graph (Bullmore & Sporns, 2009) defined as a set of nodes and edges representing the structural or functional relationship between two nodes. Brain networks analyzed using graph theory provide comprehensive information about the dynamic interactions among multiple brain regions in many neurologic disorders, including dementia.

In the current study, we measured the WM network using graph theory applied to diffusion tensor imaging (DTI) and investigated the relationship among AD, LBD, WM network disruption, and cognitive dysfunction. We hypothesized that both AD and LBD would independently affect global network measures and local network measures with disease‐specific regional patterns.

## 3 METHODS

### 3.1 Participants

We enrolled cognitively impaired patients who had either AD or LBD and control subjects from November 2015 to September 2017 from the dementia and movement clinics of Yonsei University Severance Hospital, Seoul, Korea. Patients with mild cognitive impairment (MCI) or dementia were simultaneously enrolled to identify brain changes related with early disease stage. All patients underwent neurologic examination, neuropsychological tests, and 3‐Tesla MRI. Clinical features of LBD including parkinsonism, rapid eye movement (REM) sleep behavior disorder (RBD), visual hallucination, and cognitive fluctuation were evaluated using semi‐structured questionnaires administered by caregivers. The severity of parkinsonism was assessed according to the Movement Disorder Society Unified Parkinson's Disease Rating Scale (UPDRS) motor score and was regarded as moderate if the score was > 16.

All AD patients fulfilled the criteria for probable AD dementia with high levels of biomarker evidence (McKhann et al., 2011), and all MCI due to AD patients met the criteria for high likelihood of MCI due to AD from the National Institute on Aging‐Alzheimer's Association workgroups guidelines for AD (Albert et al., 2011). All these patients were identified as having significant cerebral β‐amyloid deposition on ^18^F‐Florbetaben (FBB) PET scans and regarded to have AD‐related cognitive impairment (ADCI). PD and DLB patients were recruited using the United Kingdom PD Brain Bank diagnostic criteria (Gibb & Lees, 1988) and the 2017 revised criteria for DLB (McKeith et al., 2017), respectively. PD‐MCI and PD dementia (PDD) were diagnosed according to the level II PD‐MCI criteria and clinical criteria of probable PDD, respectively (Emre et al., 2007; Litvan et al., 2012). All patients with MCI due to DLB met the probable DLB criteria except for the presence of dementia (McKeith et al., 2017). All PD and DLB patients were confirmed to have dopaminergic depletion on ^18^F‐N‐fluoropropyl‐2b‐carbomethoxy‐3b‐(4‐iodophenyl) nortropane (FP‐CIT) PET scans and comprised the Lewy body‐related cognitive impairment (LBCI) group. All LBCI patients also underwent FBB PET scans, and if they had significant cerebral β‐amyloid deposition, they were regarded to have simultaneous ADCI. As a result, 22 patients with pure ADCI, 19 patients with pure LBCI, and 36 patients with mixed ADCI/LBCI were recruited.

Control subjects were recruited through poster advertisements for healthy older adults visiting the Yonsei University Medical Center (Institutional Review Board No. 4‐2015‐0551). They did not have any subjective symptoms of cognitive impairment or a history of neurologic or psychiatric illnesses. All 37 controls had normal cognitive function according to the Korean version of the Mini‐Mental State Examination (K‐MMSE) and detailed neuropsychological tests (described below) and exhibited normal findings on neurologic examination, structural MRI, ^18^F‐fluorodeoxyglucose PET, and FBB PET.

Exclusion criteria were the following: (1) suspected non‐AD pathophysiology; (2) pure vascular cognitive impairment; (3) other degenerative causes of dementia, including frontotemporal dementia, corticobasal degeneration, and progressive supranuclear palsy; (4) drug‐induced cognitive impairment; and (5) other causes sufficiently explaining cognitive impairment, including epilepsy, psychiatric disorder, normal pressure hydrocephalus, and structural brain lesion (e.g., tumor or hemorrhage).

This study was approved by the Institutional Review Board of the Yonsei University Severance Hospital. Informed consent was obtained from all participants.

### 3.2 Neuropsychological test

All study participants underwent the Seoul Neuropsychological Screening Battery and standardized z scores were available for all scorable tests based on age‐ and education‐matched norms(Y. Kang et al., 2003). The scorable tests were considered abnormal when the scores were below −1.0 *SD* of the norms of age‐ and education‐matched normal subjects. Among the scorable tests, we included the digit span backward test for the attention domain; the Korean version of the Boston Naming Test (K‐BNT) for the language domain; the copying item of the Rey–Osterrieth Complex Figure Test (RCFT copy) for the visuospatial domain; immediate recall, 20‐min delayed recall, and recognition items of the RCFT and Seoul Verbal Learning Test (SVLT) for the memory domain; and phonemic Controlled Oral Word Association Test (COWAT), semantic COWAT, and the Stroop color reading test for the frontal/executive domain. The control group had normal scores on all scorable tests.

### 3.3 Acquisition and interpretation of FBB PET and FP‐CIT‐PET scans

Detailed methods for FP‐CIT‐PET and FBB PET acquisitions have been described in a previous study (Y. G. Lee, Jeon, et al., 2018). On the basis of visual ratings by an expert reader (M.Y.) who was blinded to the clinical diagnosis, brain β‐amyloid plaque load score (Barthel et al., 2011) and FP‐CIT‐PET abnormalities were assessed (Oh et al., 2012). A brain β‐amyloid plaque load score of 1 was classified as β‐amyloid negative, and scores of 2 and 3 were classified as β‐amyloid positive.

### 3.4 Acquisition and processing of MR data

All participants were scanned using a Philips 3.0 T MR scanner (Philips Achieva; Philips Medical Systema, Best, The Netherlands) with a SENSE head coil (SENSE factor = 2).

Detailed information about the MR data processing is in the supplementary material.

### 3.5 Measurement of regional white matter hyperintensities

A visual rating scale of WM hyperintensities (WMH) was modified from the Fazekas scale. Periventricular WMH (PWMH) and deep WMH (DWMH) areas were classified according to a previously described protocol (S. Kim, Choi, et al., 2015). PWMH areas were classified as P1 (cap and band < 5 mm), P2 (5 mm ≤ cap or band < 10 mm), and P3 (10 mm ≤ cap or band); DWMH areas were classified as D1 (maximum diameter of deep white matter lesion < 10mm), D2 (10mm ≤ lesion <25 mm), and D3 (≥25 mm).

### 3.6 Network construction

Brain networks consist of nodes and edges, which are basic elements of a graph. Network nodes were defined according to our modified AAL atlas. We used the Automated Anatomical Labeling atlas (AAL) which parcellates the brain into 45 regions, including six subcortical regions from each hemisphere (Tzourio‐Mazoyer et al., 2002). Additionally, we included bilateral substantia innominata (SI) in this atlas. The SI was defined as in our previous study (Y. Lee, Ham, et al., 2018). It did not overlap the other parcellated brain regions of AAL. The whole‐brain streamlines and the modified AAL atlas were in the same diffusion native space to account for individual differences in brain parcellation (Lo et al., 2010). Network edges were evaluated as structurally connected when at least three streamlines connected a pair of nodes end‐to‐end. A threshold for the number of streamlines was selected to reduce the risk of false‐positive connections due to noise or limitations in the deterministic tractography (H. J. Kim, Im, et al., 2015; Shu et al., 2011). The fractional anisotropy (FA) value is considered an important index to evaluate fiber integrity. In this study, the mean FA value along all streamlines connecting pairs of regions, calculated by multiplying the number of streamlines, was used to weigh the edges. This means that if there were two nodes connected to the same number of streamlines, the weights of the edges would be different when the FA values were considered (Lo et al., 2010). The weight of the edges was divided by the average volume of the two brain regions for considering different node sizes. Finally, weighted structural networks were constructed as symmetric 92 × 92 matrices for individuals.

### 3.7 Network analysis

Graph theoretical analysis was performed with weighted and undirected structural networks using the Brain Connectivity Toolbox (Rubinov & Sporns, 2010) and BrainNet Viewer for visualization (Xia et al., 2013). We studied five local network measures including the nodal degree, nodal strength, local clustering coefficient, shortest path length, and betweenness centrality. In addition, we measured nine global network measures including the mean degree, mean strength, clustering coefficient, characteristic path length, global efficiency (E_glob_), normalized clustering coefficient (γ), normalized characteristic path length (λ), normalized E_glob_, and small‐worldness (σ). Normalized measures were scaled against the mean value of graph measures obtained from 100 matched random graphs that preserve the same number of nodes, edges, and degree sequence (Maslov & Sneppen, 2002).

### 3.8 Tract‐based spatial statistics analysis

Tract‐based spatial statistics (TBSS) analysis was employed to examine the relationship between WM integrity and network measures. All FA images were aligned into a common space using the nonlinear registration algorithm implemented in the TBSS package (Smith et al., 2006). The aligned FA images were averaged and thinned to create a mean FA skeleton that represented the centers of all tracts common to the group. Aligned FA data were then projected onto this skeleton by filling the skeleton with highest FA values from the nearest relevant center of streamlines. A threshold FA value of 0.2 was chosen to exclude voxels of adjacent gray matter or cerebrospinal fluid, and the resulting data were fed into a voxel‐wise cross‐subject statistical analysis.

### 3.9 Statistical analysis

Statistical analyses for demographic and clinical data were performed with the Statistical Package for the Social Sciences version 25.0 (IBM Corp., Armonk, NY). Analyses of variance and chi‐square tests were performed to compare clinical features across the disease and control groups. P under 0.05 was considered significant. Statistical analysis for network measures was assessed using the Statistical Package of MATLAB (R2017b, The MathWorks, Inc.).

Group‐wise comparisons of global and local network measures were performed using general linear models (GLM) after controlling for age, sex, education, DWMH, PWMH, and ICV. The independent and interaction effects of ADCI and LBCI on network measures were also investigated using GLMs after controlling for the same covariates. The independent effect of ADCI or LBCI was regarded as binary variable. For example, the independent effect of ADCI was considered to exist if the patient had ADCI. We included interaction terms (ADCI x LBCI) to find any significant synergistic or negative interaction: the mixed disease group had 1, while pure ADCI, pure LBCI, and control groups had 0. If the interaction terms of ADCI and LBCI were significant, ADCI, LBCI, and ADCI x LBCI were simultaneously entered as predictors. If the interaction terms were not significant, the Akaike Information Criteria (AIC) was used to select the better‐fitted model among models with interaction terms and those without (Table S1). Since the interaction terms were not significant for all analyses for global and local measures, and the models without interaction terms had lower AIC values than those with, all analyses for local and global network measures included ADCI and LBCI as predictors but not ADCI x LBCI. In the GLM analyses for local measures, false discovery rate (FDR) correction was used to correct for multiple statistical tests across the 92 nodes. FDR‐corrected P under 0.05 was considered significant.

To identify the regional WM disintegration underlying the disease‐related local network changes, voxel‐wise TBSS GLMs on skeletonized FA values were performed using the local network changes as predictors after controlling for age, sex, education, DWMH, PWMH, and ICV. For the local network measures that showed group‐level differences, TBSS GLMs were performed in the groups that showed significant differences, whereas for the local network measures that showed independent disease effect, TBSS GLMs were performed for all study participants. We employed a nonparametric permutation test where generating the null distribution was built up over 5,000 permutations. Threshold‐free cluster enhancement with the 2D parameter settings was applied to avoid an arbitrary threshold of an initial cluster formation (Smith & Nichols, 2009). Multiple comparison issues were adjusted for family‐wise error (FWE) and FWE‐corrected P under 0.05 was considered significant.

After finding the global and local network measures where there were significant disease effects or group‐level differences, we performed GLMs for neuropsychological test scores to determine the effects of the network measures on cognitive dysfunction, using the global and local network measures as predictors after controlling for age, sex, education, DWMH, PWMH, and ICV (Model 1). If ADCI or LBCI had significant independent effects on the network measures, we used an alternative statistical model which additionally controlled for the presence of ADCI or LBCI to avoid spurious association due to the presence of the disease (Model 2). GLMs were performed in the specific groups when the network measures showed group‐level differences, or in the overall study participants, when the network measures showed independent disease effects. The FDR correction was used to correct for multiple statistical tests across 14 neuropsychological tests, and FDR‐corrected P under 0.05 was considered significant.

## 4 RESULTS

### 4.1 Demographics and clinical characteristics

The demographics and clinical characteristics of the participants are shown in Table 1. The mean age was 73.36 ± 8.44 years, mean level of education was 10.51 ± 4.62 years, and mean disease duration was 3.34 ± 1.99 years in the disease group. Forty‐three of the 77 participants in the disease group were female (55.8%). There were no significant differences in sex, education, or disease duration. Participants in the pure LBCI and mixed disease groups were older than those in the control and pure ADCI groups (*p* = .012). The three disease groups had more severe PWMH and DWMH than did the control group, but there were no significant differences in the severity of WMH between them. Six of 22 patients in the pure ADCI group (27.3%), nine of 19 patients in the pure LBCI group (47.4%), and 26 of 36 patients in the mixed disease group (72.2%) had dementia. The proportion of patients with dementia was higher in the mixed disease group than in the pure ADCI group. The proportions of patients with parkinsonism, RBD, and cognitive fluctuation were higher in the mixed disease and pure LBCI groups than in the pure ADCI group. The mixed disease group had a higher proportion of patients with visual hallucinations than did the pure ADCI group. The mixed disease and pure LBCI groups had higher UPDRS motor scores than did the pure ADCI group. The Clinical Dementia Rating Sum of Boxes (CDR‐SOB) score was higher and K‐MMSE score was lower in the mixed disease group than in the pure ADCI and pure LBCI groups.

**TABLE 1:** Demographics and clinical characteristics

	Control	Pure ADCI	Pure LBCI	Mixed disease	*p* value^a^	*p* value^b^
Number	37	22	19	36		
Age, years	70.2 ± 5.6	69.8 ± 9.1^d,e,f^	75.4 ± 8.8^c^, ^d,e,f^	74.5 ± 7.3^c^, ^d,e,f^	.012	.060
Female, *n* (%)	25 (67.6)	14 (63.6)	12 (63.2)	17 (47.2)	.319	.361
Education, years	11.6 ± 4.7	10.9 ± 3.7	9.4 ± 4.6	10.9 ± 5.1	.404	.417
Disease duration, years	NA	3.9 ± 2.4	2.6 ± 1.1	3.4 ± 2.0	NA	.129
WMH scales						
Periventricular WHM	1.1 ± 0.3	1.5 ± 0.7^c^	1.8 ± 0.8^c^	1.8 ± 0.6^c^	<.001	.376
Deep WMH	1.1 ± 0.3	1.6 ± 0.6^c^	1.5 ± 0.6^c^	1.5 ± 0.4^c^	.001	.710
Cognitive status, *n* (%)					NA	.003
Non‐demented	NA	16 (72.7)^d,e,f^	10 (52.6)	10 (27.8)^d,e,f^		
Demented	NA	6 (27.3)^d,e,f^	9 (47.4)	26 (72.2)^d,e,f^		
LBD features, *n* (%)						
Moderate parkinsonism	NA	4 (18.2)^d,e,f^	18 (94.7)^d,e,f^	32 (88.9)^d,e,f^	NA	<.001
RBD	NA	0 (0)^d,e,f^	13 (68.4)^d,e,f^	18 (50.0)^d,e,f^	NA	<.001
Visual hallucination	NA	0 (0)^d,e,f^	4 (21.1)	10 (27.8)^d,e,f^	NA	.027
Fluctuation	NA	0 (0)^d,e,f^	9 (47.4)^d,e,f^	15 (41.7)^d,e,f^	NA	.001
UPDRS motor score	NA	0.59 ± 1.7^d,e,f^	20.9 ± 9.4^d,e,f^	26.6 ± 15.5^d,e,f^	NA	<.001
CCSIT score	NA	7.77 ± 2.2 ^d,e,f^	6.11 ± 2.87	5.03 ± 2.81^d,e,f^	NA	.001
CDR‐SOB	0 (0)	2.4 ± 1.2^c^, ^d,e,f^	3.1 ± 2.1^c^, ^d,e,f^	5.7 ± 3.3^c^, ^d,e,f^	<.001	<.001
K‐MMSE	28.6 ± 1.2	22.9 ± 3.1^c^, ^d,e,f^	23.2 ± 3.5^c^, ^d,e,f^	20.3 ± 4.9^c^, ^d,e,f^	<.001	.016

Note

Data are expressed in mean ± standard deviation or number (%). Group comparisons were performed using chi‐square tests or analyses of variance as appropriates. *p* < .05 was considered significant.

Abbreviations: ADCI, Alzheimer's disease‐related cognitive impairment; CDR‐SOB, clinical dementia rating sum of boxes; K‐MMSE, Korean version of mini‐mental state examination; LBCI, Lewy body‐related cognitive impairment; LBD, Lewy body disease; LSD, Least significant difference; NA, not applicable; RBD, REM sleep behavior disorder; UPDRS, unified Parkinson's disease rating scale; WMH, white matter hyperintensities.

^a^ Results of comparisons including the control group.

^b^ Results of comparisons between the disease groups (excluding the control group).

^c^ Significantly different in the comparison with the control group after Fisher's LSD post hoc tests.

^d,e,f^ Significantly different in the comparison between the disease groups after Fisher's LSD post hoc tests.

### 4.2 Global network measures

Group‐wise comparisons of global network measures showed that the mixed disease group had an increased λ and a decreased normalized E_glob_ compared with those values in the control group but statistically not significant (Table S2). When the interaction and independent effects of ADCI and LBCI were investigated, there were no significant interaction effects of ADCI and LBCI on global network measures. The presence of LBCI was associated with an increased λ (*p* = .042) and a decreased normalized E_glob_ (*p* = .049), independent of ADCI (Table 2).

**TABLE 2:** Effects of ADCI and LBCI on global network measures

Global measures	ADCI effect		LBCI effect	
	Beta (SE)	*p* value	Beta (SE)	*p* value
Mean degree	0.02 (0.29)	.939	−0.54 (0.29)	.070
Mean strength	0.003 (0.003)	.199	2.79 * 10^–4^ (0.003)	.914
C	2.06 * 10^–5^ (5.95 * 10^–5^)	.730	5.04 * 10^–6^ (6.02 * 10^–5^)	.934
L	−16.21 (24.34)	.507	38.12 (24.65)	.125
E_glob_	1.31 * 10^–4^ (1.49 * 10^–4^)	.382	7.06 * 10^–5^ (1.51 * 10^–4^)	.642
γ	−0.01 (0.05)	.803	0.05 (0.05)	.341
λ	0.04 (0.04)	.306	0.07 (0.04)	.042
Normalized E_glob_	−0.01 (0.01)	.215	−0.02 (0.01)	.049
σ	−0.05 (0.05)	.310	−0.08 (0.05)	.136

Note

Data are results of general linear models for global network measures after controlling for age, sex, education, intracranial volume, deep WMH, and periventricular WMH. *p* < .05 was considered significant.

Abbreviations: ADCI, Alzheimer's disease‐related cognitive impairment; C, clustering coefficient; E_glob_, global efficiency; L, characteristic path length; LBCI, Lewy body‐related cognitive impairment;SE, standard error; γ, normalized clustering coefficient; λ, normalized characteristic path length; σ, small‐worldness.

### 4.3 Local network measures

**FIGURE 1:**
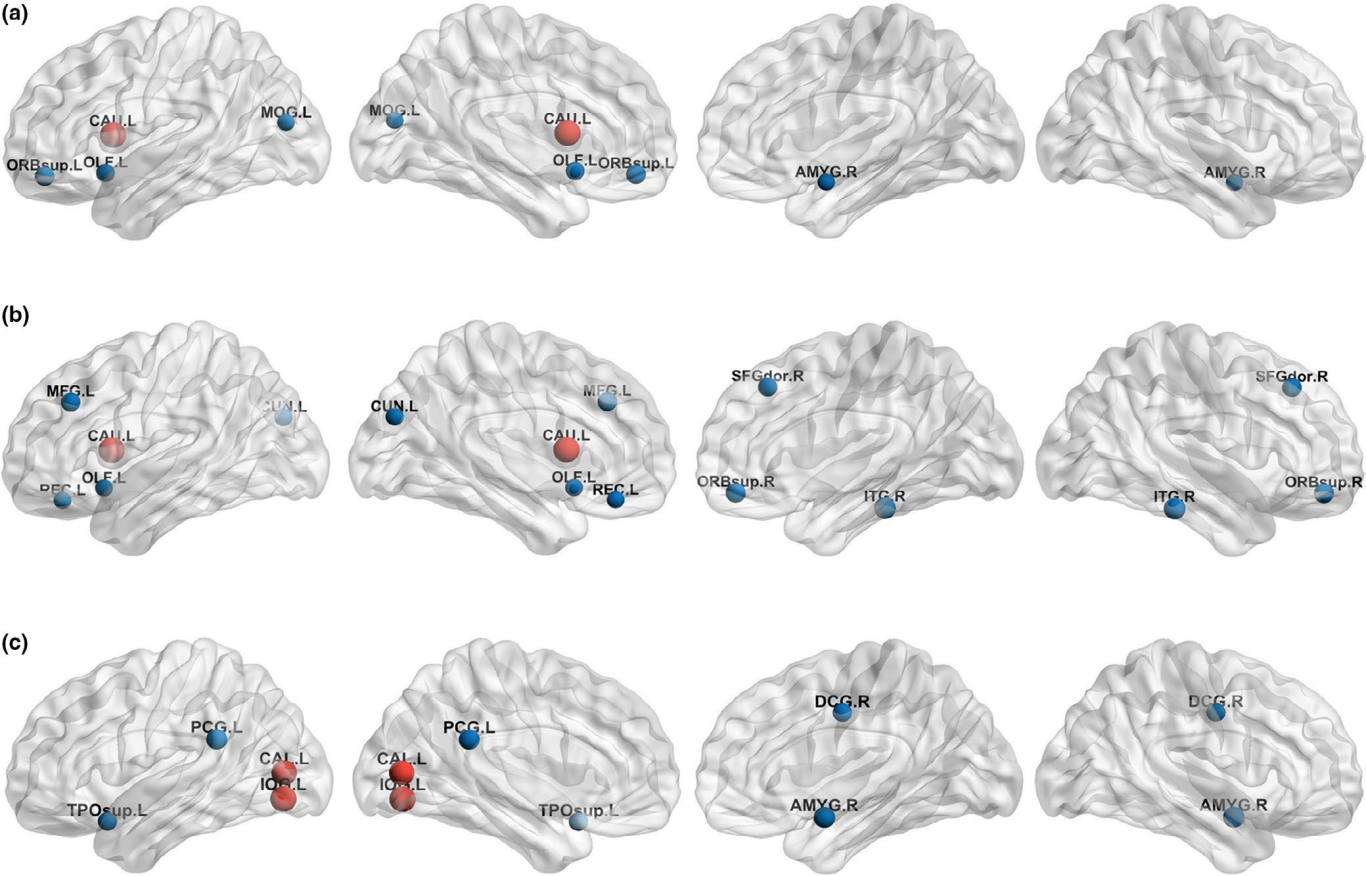
Disease‐related changes in local network measures. Disease‐related local network changes in terms of nodal degree (a, b) and betweenness centrality (c). Group‐wise comparisons of local network measures showed several nodes where the mixed disease group had lower nodal degree than the control group (a) and those where the pure LBCI group had a higher betweenness centrality than the control group (c). The presence of LBCI was negatively associated with regional nodal degree being independent of ADCI (b). ADCI was not independently associated with local network measures being independent of LBCI. Red colored nodes indicate brain nodes where FDR‐corrected P values were significant, while blue colored nodes indicate brain nodes where uncorrected P values were significant. The size of nodes represents absolute t‐value. FDR‐corrected *p* <.05 was considered significant. Abbreviations: ADCI, Alzheimer's disease‐related cognitive impairment; AMYG, Amygdala; CAL, Calcarine fissure and surrounding cortex; CAU, Caudate nucleus; CUN, Cuneus; DCG, Middle cingulate and paracingulate gyri; FDR, false discovery rate; IOG, Inferior occipital gyrus; ITG, Inferior temporal gyrus; L, Left; LBCI, Lewy body‐related cognitive impairment; MFG, Middle frontal gyrus; MOG, Middle occipital gyrus; OLF, Olfactory cortex; ORBsup, Superior frontal gyrus, orbital part; PCG, Posterior cingulate gyrus; R, Right; REC, Gyrus rectus; SFGdor, Superior frontal gyrus, dorsolateral; TPOsup, Temporal pole, superior temporal gyrus; WMH, white matter hyperintensities

Group‐wise comparisons of local network measures showed that the mixed disease group had a lower nodal degree in the left caudate than did the control group (FDR‐corrected *p* = .033) (Figure 1a), and the pure LBCI group had an increased betweenness centrality in the left calcarine cortex (FDR‐corrected *p* = .044) and left inferior occipital gyrus (FDR‐corrected *p* = .044) compared with those in the control group (Figure 1c) (Table S3). When the interaction and independent effects were investigated, there were no significant interaction effects of ADCI and LBCI on local network measures. The presence of LBCI was negatively associated with the degree of the left caudate (FDR‐corrected *p* = .033) (Figure 1b), independent of the presence of ADCI, but the independent effects of ADCI and LBCI on the betweenness centrality were not significant.

**FIGURE 2:**
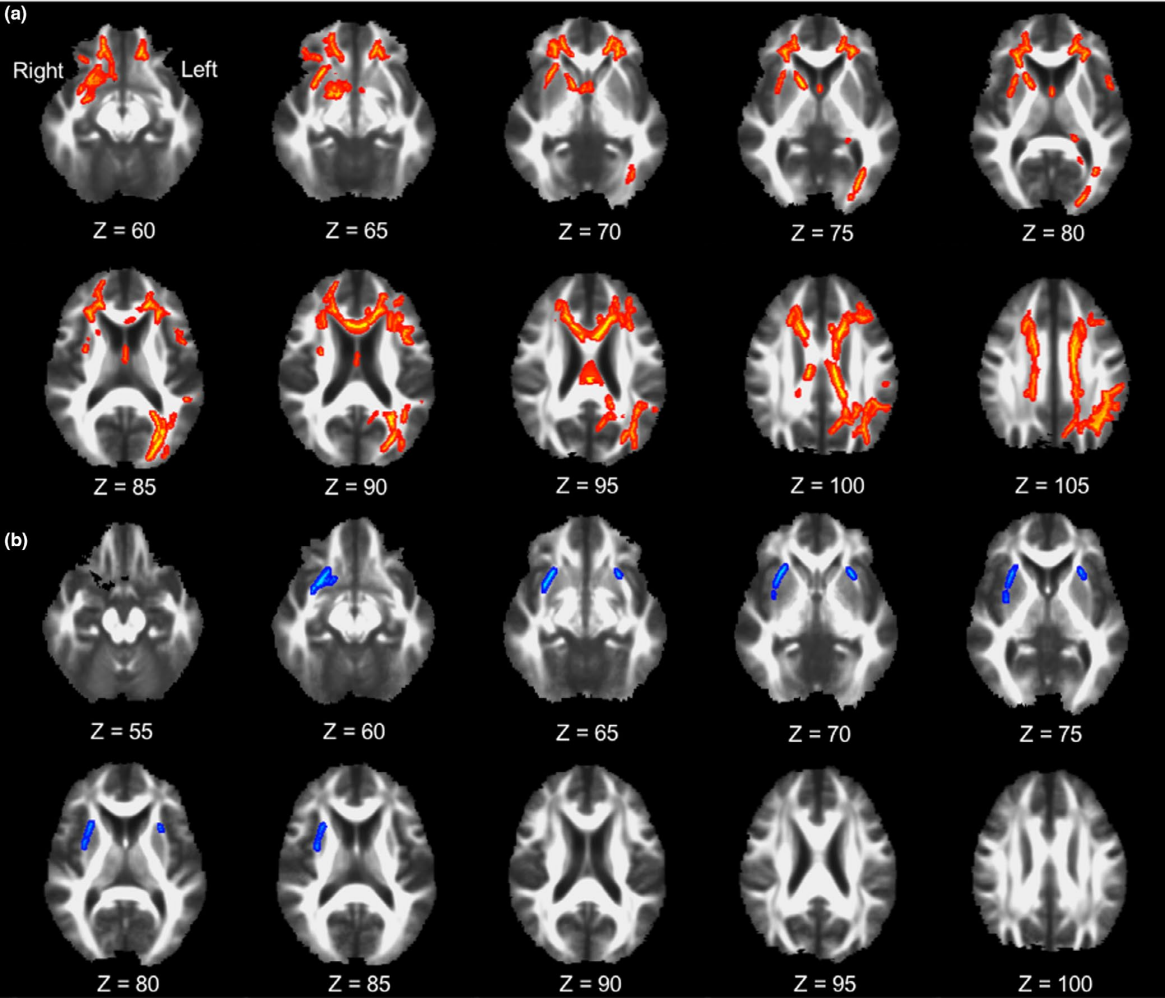
White matter disruptions correlating with the local network measures. Regional white matter disruption correlating with the left caudate nodal degree (a) and the betweenness centrality in the left inferior occipital gyrus (b). Voxel‐wise TBSS GLMs on FA skeleton using the left caudate nodal degree as a predictor were performed in overall study participants (a), while those using the betweenness centrality in the left occipital gyrus as a predictor were performed in the combined group of control and pure LBCI. Red colored voxels indicate positive correlation and blue colored voxels indicate negative correlation. Abbreviations: FA, Fractional anisotropy; GLM, general linear model; LBCI, Lewy body‐related cognitive impairment; TBSS, tract‐based spatial statistics

To determine the regional WM changes explaining the LBCI‐related decrease in left caudate nodal degree, TBSS GLMs for regional FA values were performed using the left caudate nodal degree as a predictor in the study participants overall (Figure 2a). The left caudate nodal degree was positively associated with regional FA values in the WM adjacent to the right caudate nucleus, right putamen, right amygdala, left occipital cortex, left parietal cortex, and bilateral basal frontal cortices in addition to the bilateral corpus callosum and cingulum.

To identify the regional WM changes explaining the increased betweenness centrality in the pure LBCI group compared with that in the control group, TBSS GLMs for regional FA values were performed using the betweenness centrality of the left calcarine cortex and left inferior occipital gyrus as predictors after combining the pure LBCI and control groups (Figure 2b). The betweenness centrality of the left inferior occipital gyrus was negatively associated with regional FA values in the WM adjacent to the right caudate nucleus and bilateral putamen. There was no significant correlation between FA and the betweenness centrality of the left calcarine cortex.

### 4.4 Correlation of global and local network measures with neuropsychological test scores

GLMs for neuropsychological test scores using the λ as a predictor showed that the λ was negatively associated with the scores on the K‐MMSE and all subtests of the SNSB except for the RCFT copy, RCFT immediate recall, and RCFT recognition (Table 3). After further controlling for the presence of LBCI, λ was negatively associated with the scores on the K‐MMSE, SVLT immediate recall, SVLT delayed recall, SVLT recognition, COWAT animal, COWAT supermarket, and Stroop color reading. GLMs using the normalized E_glob_ as a predictor showed that the normalized E_glob_ was positively associated with the scores on the K‐MMSE, K‐BNT, SVLT immediate recall, SVLT delayed recall, COWAT animal, COWAT supermarket, COWAT phonemic, and Stroop color reading. After further controlling for the presence of LBCI, normalized E_glob_ showed no significant relationship with neuropsychological test scores.

**TABLE 3:** Correlation of global network measures and neuropsychological test scores

	Model 1				Model 2			
	λ		Normalized E_glob_		λ		Normalized E_glob_	
	Beta (SE)	*p* value	Beta (SE)	*p* value	Beta (SE)	*p* value	Beta (SE)	*p* value
Digit span backward	−1.66 (0.77)	.033^a^	3.79 (2.36)	.111	−1.19 (0.76)	.122	2.38 (2.33)	.310
K‐BNT	−2.57 (0.95)	.008^a^	7.19 (2.92)	.015^a^	−1.89 (0.92)	.044	5.24 (2.83)	.067
RCFT copy	−2.52 (1.26)	.048	5.48 (3.87)	.140	−1.56 (1.23)	.205	2.78 (3.75)	.460
SVLT immediate recall	−2.58 (0.77)	.001^a^	8.26 (2.33)	.001^a^	−1.75 (0.70)	.014^a^	5.78 (2.12)	.008
SVLT delayed recall	−3.00 (0.87)	.001^a^	9.15 (2.65)	.001^a^	−2.16 (0.81)	.009^a^	6.62 (2.47)	.009
SVLT recognition	−2.79 (0.94)	.004^a^	6.18 (2.90)	.036	−2.12 (0.92)	.023^a^	4.03 (2.82)	.156
RCFT immediate recall	−1.25 (0.66)	.060	2.52 (2.01)	.212	−0.85 (0.65)	.193	1.28 (1.99)	.521
RCFT delayed recall	−1.60 (0.70)	.025^a^	4.29 (2.14)	.048	−1.23 (0.70)	.083	3.14 (2.14)	.146
RCFT recognition	−0.70 (0.84)	.406	2.08 (2.55)	.418	−0.22 (0.84)	.796	0.62 (2.54)	.806
COWAT animal	−2.46 (0.65)	<.001^a^	6.11 (2.03)	.003^a^	−0.19 (0.63)	.003^a^	4.55 (1.95)	.022
COWAT supermarket	−2.03 (0.59)	.001^a^	5.16 (1.83)	.006^a^	−1.63 (0.58)	.006^a^	3.97 (1.78)	.028
COWAT phonemic	−1.70 (0.61)	.006^a^	4.40 (1.89)	.022^a^	−1.07 (0.56)	.057	2.58 (1.71)	.135
Stroop color reading	−3.70 (0.81)	<.001^a^	7.91 (2.62)	.003^a^	−2.84 (0.74)	<.001^a^	5.38 (2.35)	.024
K‐MMSE	−8.66 (2.35)	<.001^a^	20.68 (7.31)	.006^a^	−6.47 (2.21)	.004^a^	13.81 (6.85)	.047

Note

Data are results of general linear models for standardized neuropsychological test scores using global network measures as predictors. Covariates included age, sex, education, intracranial volume, deep WMH, and periventricular WMH for statistical models (model1). The presence of LBCI was further controlled for model 2.

Abbreviations: COWAT, controlled oral word association test; E_glob_, global efficiency; FDR, false discovery rate; K‐BNT, Korean version of the Boston naming test; RCFT, Rey–Osterrieth complex figure test; SE, standard error; SVLT, Seoul verbal learning test; WMH, white matter hyperintensities; λ, normalized characteristic path length.

^a^ FDR‐corrected *p* <.05 that were performed for multiple statistical tests across 14 neuropsychological tests.

In the study participants overall, lower left caudate nodal degree was associated with lower scores in all neuropsychological tests except for the immediate and delayed recall items of the RCFT (Table 4). After further controlling for the presence of LBCI, the left caudate nodal degree was positively associated with the scores on the K‐MMSE and RCFT recognition. In the combined pure LBCI and control groups, the increased betweenness centrality of the left calcarine cortex was associated with lower scores in all neuropsychological tests except for the RCFT copy and RCFT recognition, whereas the increased betweenness centrality of the left inferior occipital gyrus was associated with all neuropsychological tests except for the digit span backward and RCFT recognition. After further controlling for the presence of LBCI, the betweenness centrality of the left calcarine cortex was negatively associated with the scores on the K‐BNT and SVLT delayed recall, whereas the increased betweenness centrality of the left inferior occipital gyrus was associated with the scores on the K‐MMSE and RCFT copy.

**TABLE 4:** Correlation of local network measures and neuropsychological test scores

	Left caudate nodal degree				Left calcarine BC				Left inferior occipital BC			
	Model 1		Model 2		Model 1		Model 2		Model 1		Model 2	
	Beta (SE)	*p* value	Beta (SE)	*p* value	Beta (SE)	*p* value	Beta (SE)	*p* value	Beta (SE)	*p* value	Beta (SE)	*p* value
Digit span backward	0.07 (0.03)	.007^a^	0.04 (0.03)	.092	−8.33 (3.26)	.014^a^	−7.37 (3.75)	.056	−4.19 (13.60)	.760	8.28 (15.24)	.590
K‐BNT	0.10 (0.03)	.002^a^	0.07 (0.03)	.042	−19.65 (3.40)	<.001^a^	−15.13 (3.68)	<.001^a^	−42.49 (16.26)	.012 ^a^	−14.28 (16.70)	.397
RCFT copy	0.11 (0.04)	.007^a^	0.06 (0.04)	.137	−6.38 (3.29)	.058	−1.56 (3.38)	.648	−48.58 (11.46)	<.001^a^	−35.24 (12.46)	.007^a^
SVLT immediate recall	0.09 (0.03)	<.001^a^	0.05 (0.02)	.046	−10.28 (3.15)	.002^a^	−3.18 (2.73)	.250	−28.27 (13.07)	.036 ^a^	4.38 (11.01)	.693
SVLT delayed recall	0.10(0.03)	<.001^a^	0.05 (0.03)	.059	−17.30 (2.84)	<.001^a^	−11.77 (2.67)	<.001^a^	−31.38 (14.17)	.032^a^	1.37 (12.63)	.914
SVLT recognition	0.08 (0.03)	.013^a^	0.04 (0.03)	.216	−12.12(2.78)	<.001^a^	−6.19 (2.50)	.017	−38.61 (11.66)	.003^a^	−11.78 (10.43)	.265
RCFT immediate recall	0.03 (0.02)	.233	0.002 (0.02)	.936	−5.86 (2.78)	.040^a^	−3.31 (3.05)	.283	−25.48 (10.85)	.023 ^a^	−15.74 (12.04)	.197
RCFT delayed recall	0.04 (0.02)	.061	0.02 (0.02)	.353	−6.89 (2.75)	.016^a^	−4.02 (2.98)	.184	−27.68 (10.80)	.014 ^a^	−16.14 (11.83)	.179
RCFT recognition	0.11 (0.03)	<.001^a^	0.09 (0.03)	.001^a^	−4.14 (3.34)	.222	−0.39 (3.59)	.915	−23.19 (12.94)	.079	−9.66 (14.21)	.500
COWAT animal	0.05 (0.02)	.017^a^	0.02 (0.02)	.325	−6.58 (3.10)	.039^a^	−2.74 (3.39)	.423	−31.51 (11.86)	.011^a^	−18.24 (13.08)	.170
COWAT supermarket	0.06 (0.02)	.004^a^	0.04 (0.02)	.074	−7.85 (2.63)	.004^a^	−5.24 (2.93)	.080	−25.29 (10.59)	.021^a^	−13.44 (11.68)	.256
COWAT phonemic	0.06 (0.02)	.005^a^	0.02 (0.02)	.247	−8.33 (2.29)	<.001^a^	−5.55 (2.51)	.032	−22.25 (9.62)	.025 ^a^	−8.21 (10.25)	.428
Stroop color reading	0.10 (0.03)	<.001^a^	0.05 (0.03)	.071	−11.20 (3.08)	<.001^a^	−5.44 (3.09)	.085	−45.76 (11.89)	<.001^a^	−23.85 (12.00)	.053
K‐MMSE	0.34 (0.07)	<.001^a^	0.23 (0.08)	.003^a^	−22.58 (7.45)	.004^a^	−7.37 (6.87)	.289	−164.17 (21.50)	<.001^a^	−122.96 (21.02)	<.001^a^

Note

Data are results of general linear models for standardized neuropsychological test scores using local network measures. Analysis using the nodal degree was performed in overall study participants, while those using the betweenness centrality was performed in the combined group of control and pure LBCI. Covariates included age, sex, education, intracranial volume, deep WMH, and periventricular WMH for statistical models (model1). The presence of LBCI was further controlled for model 2.

Abbreviations: BC, betweenness centrality; COWAT, controlled oral word association test; FDR, false discovery rate; K‐BNT, Korean version of the Boston naming test; RCFT, Rey–Osterrieth complex figure test; SE, standard error; SVLT, Seoul verbal learning test; WMH, white matter hyperintensities.

^a^ FDR‐corrected *p* < .05 that were performed for multiple statistical tests across 14 neuropsychological tests.

## 5 DISCUSSION

In this study, we investigated the WM network changes related to ADCI and LBCI and their effects on cognitive dysfunction. Our major findings are as follows. First, LBCI, especially mixed disease with ADCI, was associated with disintegration of global network measures, reflected in the increased λ and decreased normalized E_glob_, which in turn were associated with cognitive dysfunction. Second, LBCI was associated with decreased nodal degree in the left caudate, which was associated with decreased FA in the brain regions vulnerable to LBD and K‐MMSE. Third, compared with the control group, the pure LBCI group had an increased betweenness centrality in the left inferior occipital gyrus, which was associated with decreased FA in the WMs adjacent to the bilateral striatum and visuospatial dysfunction. Taken together, our findings suggest that concomitant ADCI and LBCI are associated with the accentuation of LBCI‐related WM network disruption centered in the left caudate nucleus, and occipital increase of betweenness centrality could be a characteristic biologic change associated with visuospatial dysfunction in pure LBCI.

To the best of our knowledge, this study is the first to investigate WM network disruption while simultaneously considering AD and LBD using graph theory‐based DTI network measures. We showed that LBCI, but not ADCI, was independently associated with global WM network measures including increased λ and decreased normalized E_glob_. In contrast to our findings, a previous study comparing graph theory‐based WM network between AD patients and control subjects showed that AD was related to increased λ and reduced global efficiency (Lo et al., 2010). However, another study that evaluated the effects of β‐amyloid deposition and vascular MRI markers on global network measures in patients with AD or subcortical vascular dementia found that β‐amyloid deposition did not affect global network measures, but vascular MRI markers did (H. J. Kim, Im, et al., 2015). Therefore, our results suggest that functional integration (Rubinov & Sporns, 2010) through structural WM connectivity is more vulnerable to LBD than AD.

However, it is noteworthy that group differences of λ and normalized E_glob_ were significant in the comparison between the mixed disease and control groups, but not in the comparison between the pure LBCI and control groups. Although the interaction effects of ADCI and LBCI on global network measures were not significant, mixed AD and LBD could induce prominent network disruption, and the independent effect of LBCI on global network disruption was mainly driven by the mixed disease group. This point of view is consistent with our results that λ and normalized E_glob_ correlated with neuropsychological test scores typically affected by AD (memory), LBD (Stroop color reading test), and both diseases (semantic fluency). Considering that λ and normalized E_glob_ measure the ability to combine or transfer information between distant brain regions (Rubinov & Sporns, 2010), and they correlate with intelligence or cognitive performance in healthy subjects (Li et al., 2009), our results highlight the importance of mixed LBD and AD in cognitive deterioration and global WM network disruption in the spectrum from healthy aging to dementia.

Our second major finding was that LBCI was independently associated with decreased nodal degree in the left caudate nucleus, where the mixed disease group had significantly lower nodal degree than did the control group. The caudate nucleus is a brain region where converging evidence suggests nigrostriatal dopaminergic depletion (O'Brien et al., 2004; Piggott et al., 1999), brain atrophy (Barber et al., 2002), and DTI abnormalities (Bozzali et al., 2005) in DLB patients. The caudate nucleus is also a prominent subcortical region of β‐amyloid deposition in LBD (Kalaitzakis et al., 2008) as well as in AD patients (Hanseeuw et al., 2018). Considering that transmission of α‐synuclein is a key biologic change in LBD (Irwin et al., 2013), and β‐amyloid peptides enhance α‐synuclein accumulation and neuronal deficits in a transgenic mouse model (Masliah et al., 2001), the caudate nucleus could be the core site of synergistic interaction between LBD and AD pathologies. Decreased left caudate nodal degree correlated with a regional FA decrease across widespread WM regions and global cognitive dysfunction in the study participants overall. The topography of FA decrease related to left caudate nodal degree is in agreement with previously reported FA decreases observed in the corpus callosum, frontal WM, and parietal WM of PD and DLB patients (Agosta et al., 2014; Galantucci et al., 2017; Lee et al., 2010), and the occipital WM in DLB patients (Bozzali et al., 2005; Lee et al., 2010). Therefore, our results suggest that the left caudate nucleus could be the center of widespread WM network disruption in LBD patients.

Our third major finding was that the pure LBCI group had an increased betweenness centrality in the left calcarine cortex and left inferior occipital gyrus compared with those of the control group, and the increased betweenness centrality of the left inferior occipital gyrus correlated with the decreased FA values of the WMs adjacent to the striatum. Our result is in line with that of a recent study that investigated the metabolic brain network differences between DLB patients and control subjects and revealed that DLB patients have an increased betweenness centrality in the occipital nodes (Chen et al., 2018). As increased betweenness centrality in the left occipital gyrus was associated with striatal FA decrease and the increased betweenness centrality in the left inferior occipital gyrus had the strongest association with visuospatial dysfunction in our study, our results could be interpreted as an epiphenomenon rather than a causal relationship. Cortical connections from the striatum are more focused in the frontal and parietal cortices than in the occipital cortex (Cacciola et al., 2017), and frontoparietal cortices are involved in visuospatial function in humans (Drag et al., 2016). Although still a speculation, LBD‐related WM disconnection between the striatum and frontoparietal cortices could deteriorate information processing between the frontal and parietal cortices via the striatum, and the less affected bypass through the fronto‐occipital and occipito‐parietal connections could become more important. In this paradigm, the more LBD‐related striatal WM disruption progresses, the more occipital betweenness centrality increases and visuospatial dysfunction deteriorates.

This study has several limitations. First, because of its cross‐sectional design, this study could not determine the causality of LBCI on WM change. Future longitudinal studies are warranted. Second, patients who mainly exhibited AD features and mild parkinsonism and those who mainly had LBD features and positive amyloid PET results were uniformly regarded as the mixed disease group. Furthermore, the effects of ADCI and LBCI could differ according to the main clinical symptoms. Future studies with a larger sample size are needed to test this issue. Third, we did not perform corrections for multiple statistical tests across nine global network measures in the analyses for the effects of ADCI and LBCI on global network measures. However, we did not want to miss the possible associations between network measures and AD or LBD in the early stage of analyses in this explorative study. Future confirmatory studies with larger sample size are warranted to confirm our results. Forth, due to the small sample size in each subgroup, especially in the pure LBCI group, we could not evaluate the patterns of correlation in each subgroup. Future studies with larger sample sizes are needed to determine the effect of network disruption on the clinical severity of each disease. Fifth, there is no biomarker surrogate to measure LB pathology, and we used dichotomized approach based on dopamine transporter PET imaging and clinical assessment. It is necessary to interpret the results cautiously with a dichotomized approach for AD and LBD. In addition, participants in the pure LBCI and mixed disease groups were older than those in the control and pure ADCI groups. This difference could affect our result since the aging is related to the deterioration of microstructural integrity of WM in other studies (Sullivan & Pfefferbaum, 2006; Voineskos et al., 2012). However, we included age as a covariate in every statistical model, and the effects of age on the λ, normalized E_glob_, left caudate nodal degree, left calcarine betweenness centrality, and left inferior occipital betweenness were not significant. Finally, treating cortical nodes and subcortical nodes equally that have complex tissue structure could have biased our results, and there is no consensus method for building the most appropriate graph measures. In addition, the low b‐values used in the diffusion sequence could be a potential limitation. Further studies with more advanced diffusion weighed image sequence should confirm the results of the study. Nevertheless, our findings provide a clue for understanding the relationship between WM connectivity and the two most common degenerative causes of dementia, AD and LBD.

## 6 CONCLUSION

This result suggests that concomitant ADCI and LBCI are associated with the accentuation of LBCI‐related WM network disruption centered in the left caudate nucleus where could be the core site of synergistic interaction between LBD and AD pathologies. Moreover, the pure LBCI group had an increased betweenness centrality in the left inferior occipital gyrus, which was associated with visuospatial dysfunction. Taken together, our findings provide a better understanding about WM connectivity within AD and LBD.

## CONFLICT OF INTEREST

Nothing to report.

## AUTHOR CONTRIBUTIONS

All authors have reviewed the manuscript being submitted, approved of its contents, and validated the accuracy of the data. K.B, J.Y, J.L, and B.S.Y involved in conceptualization, design of the study, and drafting the text or preparing the figures. K.B, J.Y, J.H.J, Y.H.L, S.J.J, H.S.Y, Y.H.S, P.H.L, J.L, and B.S.Y contributed to acquisition and analysis of data.

### PEER REVIEW

The peer review history for this article is available at https://publons.com/publon/10.1002/brb3.2112.

## Supporting information

Supplementary Material

## Data Availability

The data and code used in this work will be available from the corresponding authors upon reasonable request.
