# Supplementary material for: Structural connectivity networks in Alzheimer’s disease and Lewy body disease
Source: Brain Behav. 2021 Apr 1;11(5):e02112. doi: 10.1002/brb3.2112 (PMC8119831; doi:10.1002/brb3.2112)
Supplement: Supplementary file 1 — Supplementary Material [file BRB3-11-e02112-s001.docx]

**Supplementary methods**

Acquisition and processing of MR data

T1-weighted MRI data were obtained using a 3D T1-TFE sequence with the following parameters: 224 × 224 axial acquisition matrix; 256 × 256 reconstructed matrix with 170 slices; voxel size, 0.859 × 0.859 × 1 mm^3^; field of view, 220 mm; echo time, 4.6 ms; repetition time, 9.8 ms; flip angle, 8°. With the baseline image without weighting, diffusion-weighted MRI data were acquired from 32 diffusion sampling directions using a single-shot echo-planar acquisition with the following parameters: 128 × 128 acquisition matrix with 70 slices; voxel size, 1.75 × 1.75 × 2 mm^3^; field of view, 220 mm; b-factor, 600 s/mm^2^; echo time, 70 ms; repetition time, 7.663 sec; and flip angle, 90°. Conventional 2-dimensional fluid-attenuated inversion recovery images were obtained to evaluate WMHs.

Individual T1-weighted images were co-registered to the non-diffusion-weighted (b = 0) images using affine transformation. The T1 images were linearly and non-linearly registered to the standard space of the ICBM152 T1 template to obtain the transformation matrix ^1^. This modified AAL atlas consisted of 92 brain regions in the standard space and was transformed to each individual’s non-diffusion-weighted space using inverse transformation with a nearest neighbor interpolation method. Regional gray matter volume was calculated in the modified AAL atlas to account for variations in node size. Brain tissues were classified into WM, gray matter, cerebrospinal fluid, and background using an advanced neural-net ^2^. Intracranial volume (ICV) was calculated as the sum of gray matter, WM, and cerebrospinal fluid after removing the skull ^3^.

Diffusion weighted image data were processed using the diffusion toolbox of the FSL (FMRIB’s Software Library) package for preprocessing and DSI Studio (<http://dsi-studio.labsolver.org)> for tractography. Motion artifacts and eddy current distortion were corrected by re-aligning all scans to the b0 images. Diffusion tensor matrices such as fractional anisotropy (FA) and mean diffusivity were estimated by fitting a tensor model to the raw diffusion data. Whole-brain WM fiber tracts were constructed by selecting all WM voxels as a seeding region in diffusion native space using the deterministic fiber tracking algorithm ^4^. We terminated tracking when the angle between two consecutive orientation vectors was greater than the given threshold of 45° or when the FA value was lower than 0.2. A fiber cutoff filter was applied such that fibers shorter than 20 mm and longer than 400 mm were discarded.

**Supplementary table 1. Model fitness**

|  | AIC | |
| --- | --- | --- |
| Global measures | Model with interaction terms | Model without interaction terms |
| Mean degree | 404.270 | 402.363 |
| Mean strength | -675.852 | -677.849 |
| C | -1644.536 | -1646.519 |
| L | 1414.700 | 1413.415 |
| E_glob_ | -1361.138 | -1363.085 |
| γ | -7.371 | -8.877 |
| λ | -75.909 | -77.507 |
| Normalized E_glob_ | -329.517 | -331.294 |
| σ | 6.423 | 4.494 |

Data are AIC of general linear models for global network measures after controlling for age, sex, education, intracranial volume, deep WMH, and periventricular WMH. In model with interaction terms, independent effects of ADCI, LBCI and interaction term between ADCI and LBCI were included as predictors. In model without interaction terms, independent effects of ADCI, LBCI were included as predictors.

Abbreviations: AIC, Akaike Information Criteria; ADCI, Alzheimer’s disease related cognitive impairment; C, clustering coefficient; E_glob_, global efficiency; γ, normalized E_glob_; L, characteristic path length; λ, normalized characteristic path length; LBCI, Lewy body related cognitive impairment; σ, small-worldness; WMH, white matter hyperintensities.

**Supplementary table 2. Group-wise comparison of global network measures**

| Global measures | Control | Pure ADCI | Pure LBCI | Mixed disease | P value |
| --- | --- | --- | --- | --- | --- |
| Mean degree | 17.54 (1.43) | 17.47 (1.00) | 16.54 (1.58) | 16.77 (1.39) | 0.325 |
| Mean strength | 0.07 (0.01) | 0.07 (0.01) | 0.06 (0.01) | 0.07 (0.02) | 0.617 |
| C | 0.001 (0.0003) | 0.001 (0.0004) | 0.001 (0.0002) | 0.001 (0.0003) | 0.956 |
| L | 469.83 (87.21) | 486.12 (84.48) | 571.13 (175.58) | 526.65 (136.05) | 0.371 |
| E_glob_ | 0.003 (0.001) | 0.004 (0.001) | 0.003 (0.001) | 0.003 (0.001) | 0.725 |
| γ | 2.66 (0.23) | 2.69 (0.14) | 2.80 (0.28) | 2.73 (0.22) | 0.714 |
| λ | 1.31 (0.15) | 1.37 (0.17) | 1.38 (0.16) | 1.40 (0.17)^a^ | 0.087 |
| Normalized E_glob_ | 0.89 (0.05) | 0.87 (0.05) | 0.87 (0.06) | 0.85 (0.05)^a^ | 0.079 |
| σ | 2.05 (0.25) | 1.99 (0.24) | 2.04 (0.20) | 1.97 (0.26) | 0.256 |

Data are expressed in mean **(**standard deviation). P values are results of general linear models for global network measures after controlling for age, sex, education, intracranial volume, deep WMH, and periventricular WMH. P < 0.05 was considered significant.

Abbreviations: ADCI, Alzheimer’s disease related cognitive impairment; LBCI, Lewy body related cognitive impairment; C, clustering coefficient; L, characteristic path length; Eglob, global efficiency; γ, normalized clustering coefficient; λ, normalized characteristic path length; σ, small-worldness.

^a^ Significantly different in comparisons with the control group without correction for multiple comparison.

**Supplementary table 3. Group-wise comparison of local network measures**

| Global measures | Control | Pure ADCI | Pure LBCI | Mixed disease | P value |
| --- | --- | --- | --- | --- | --- |
| Left caudate nodal degree | 21.22 (6.02) | 18.00 (4.89) | 15.95 (4.22) | 13.83 (3.61)^a,^ | < 0.001 |
| Left calcarine BC | 0.01 (0.02) | 0.03 (0.05) | 0.06 (0.08)^a^ | 0.03 (0.04) | 0.601 |
| Left inferior occipital BC | 8.78*10^-4^ (3.79 *10^-3^) | 5.29*10^-3^ (1.18 *10^-2^) | 1.09*10^-2^ (2.17*10^-2^)^a^ | 3.90*10^-3^ (9.54*10^-3^) | 0.541 |

Data are expressed in mean **(**standard deviation). P values are results of general linear models for local network measures after controlling for age, sex, education, intracranial volume, deep WMH, and periventricular WMH. P values are FDR corrected. FDR-corrected P < 0.05 was considered significant.

Abbreviations: ADCI, Alzheimer’s disease related cognitive impairment; BC, betweenness centrality; FDR, false discovery rate; LBCI, Lewy body related cognitive impairment; WMH, white matter hyperintensities.

^a^ Significantly different in comparisons with the control group.

**Supplementary references**

1. Collins DL, Neelin P, Peters TM, et al. Automatic 3d Intersubject Registration of Mr Volumetric Data in Standardized Talairach Space. *J Comput Assist Tomo* 1994;18(2):192-205.

2. Zijdenbos A, Evans A, Riahi F, et al. Automatic quantification of multiple sclerosis lesion volume using stereotaxic space. *Lect Notes Comput Sc* 1996;1131:439-48.

3. Smith SM. Fast robust automated brain extraction. *Hum Brain Mapp* 2002;17(3):143-55. doi: 10.1002/hbm.10062

4. Yeh FC, Verstynen TD, Wang Y, et al. Deterministic diffusion fiber tracking improved by quantitative anisotropy. *PLoS One* 2013;8(11):e80713. doi: 10.1371/journal.pone.0080713 [published Online First: 2013/12/19]
